# Nitrite circumvents platelet resistance to nitric oxide in patients with heart failure preserved ejection fraction and chronic atrial fibrillation

**DOI:** 10.1093/cvr/cvy087

**Published:** 2018-04-12

**Authors:** Alessandra Borgognone, Eduard Shantsila, Sophie M Worrall, Eakkapote Prompunt, Thomas Loka, Brodie L Loudon, Myriam Chimen, G Ed Rainger, Janet M Lord, Ashley Turner, Peter Nightingale, Martin Feelisch, Paulus Kirchhof, Gregory Y H Lip, Steve P Watson, Michael P Frenneaux, Melanie Madhani

**Affiliations:** 1 Institute of Cardiovascular Sciences, University of Birmingham, Edgbaston, Birmingham B15 2TT, UK; 2 Sandwell and West Birmingham NHS Trust, City Hospital, Birmingham B18 7QH, UK; 3 Norwich Medical School, University of East Anglia, Norwich NR4 7UQ, UK; 4 Institute of Inflammation and Ageing, University of Birmingham, Edgbaston, Birmingham B15 2TT, UK; 5 Wellcome Trust Clinical Research Facility, Queen Elizabeth Hospital, Edgbaston, Birmingham B15 2TT, UK; 6 Clinical and Experimental Sciences, Faculty of Medicine, University of Southampton, Southampton SO16 6YD, UK

**Keywords:** Nitrite, Platelets, Nitric oxide, Heart failure with preserved ejection fraction, Atrial fibrillation

## Abstract

**Aims:**

Heart failure (HF) is a pro-thrombotic state. Both platelet and vascular responses to nitric oxide (NO) donors are impaired in HF patients with reduced ejection fraction (HFrEF) compared with healthy volunteers (HVs) due to scavenging of NO, and possibly also reduced activity of the principal NO sensor, soluble guanylate cyclase (sGC), limiting the therapeutic potential of NO donors as anti-aggregatory agents. Previous studies have shown that nitrite inhibits platelet activation presumptively after its reduction to NO, but the mechanism(s) involved remain poorly characterized. Our aim was to compare the effects of nitrite on platelet function in HV vs. HF patients with preserved ejection fraction (HFpEF) and chronic atrial fibrillation (HFpEF–AF), vs. patients with chronic AF without HF, and to assess whether these effects occur independent of the interaction with other formed elements of blood.

**Methods and results:**

Platelet responses to nitrite and the NO donor sodium nitroprusside (SNP) were compared in age-matched HV controls (*n* = 12), HFpEF–AF patients (*n* = 29), and chronic AF patients (*n* = 8). Anti-aggregatory effects of nitrite in the presence of NO scavengers/sGC inhibitor were determined and vasodilator-stimulated phosphoprotein (VASP) phosphorylation was assessed using western blotting. In HV and chronic AF, both nitrite and SNP inhibited platelet aggregation in a concentration-dependent manner. Inhibition of platelet aggregation by the NO donor SNP was impaired in HFpEF-AF patients compared with healthy and chronic AF individuals, but there was no impairment of the anti-aggregatory effects of nitrite. Nitrite circumvented platelet NO resistance independently of other blood cells by directly activating sGC and phosphorylating VASP.

**Conclusion:**

We here show for the first time that HFpEF-AF (but not chronic AF without HF) is associated with marked impairment of platelet NO responses due to sGC dysfunction and nitrite circumvents the ‘platelet NO resistance’ phenomenon in human HFpEF, at least partly, by acting as a direct sGC activator independent of NO.

## 1. Introduction

Heart failure (HF) with preserved ejection fraction (HFpEF) accounts for ∼50% of HF cases.^1^ HFpEF is associated with morbidity and mortality close to that of HF with reduced ejection fraction (HFrEF) and there are no effective therapies.^2^^,^^3^ Both HFrEF and HFpEF are associated with impaired endothelial function and a number of studies in patients with HFrEF have demonstrated that tissue responsiveness to direct nitric oxide (NO) donors in blood vessels and platelets are diminished.^4^^,^^5^

Atrial fibrillation (AF) commonly accompanies HFpEF (up to 40%) and its presence is associated with substantial embolic risk. Although warfarin or non-vitamin K antagonist oral anticoagulants are commonly prescribed in this setting the haemorrhagic risk in these patients (mainly elderly, often with comorbidities) is high. Trials have shown that aspirin is relatively ineffective in reducing embolic risk in patients with chronic AF^6^ and additional therapies that might reduce embolic risk, particularly in those at high risk of bleeding complications from warfarin would be potentially valuable.

Recent onset AF is itself associated with platelet hyperaggregability, in part related to impaired NO signalling.^7^ It is well-established that NO is an important mediator in the regulation of vascular tone and inhibitor of platelet aggregation.^8^ These effects are predominantly mediated by the activation of soluble guanylate cyclase (sGC) and cyclic guanosine-3’, 5-monophosphate (cGMP), which subsequently activates protein kinase G and leads to phosphorylation of vasodilator-stimulated phosphoprotein (VASP).^9^

Circulating blood platelets exhibit abnormalities in patients with acute coronary syndrome (ACS), stable angina and ischaemic HFrEF, and potent antiplatelet therapy plays a pivotal role in management of patients with ACS.^10^ The phenomenon of ‘platelet NO resistance’ has been well described in these patient cohorts,^11^ but the mechanism of this diminished anti-aggregatory effect of NO in platelets remains poorly defined. However, it has been postulated that NO resistance is likely to be associated with NO scavenging by superoxide believed to be derived predominantly from circulating neutrophils.^12^ Other proposed mechanisms include, oxidation or loss of the haem moiety and/or oxidation of specific cysteine thiols of the sGC, thus resulting in an impairment of NO-induced sGC activity.^13^^,^^14^ NO resistance has also been described as an independent predictor of increased mortality and morbidity in patients with high risk ACS.^15^ An alternative route to activate the NO-sGC-cGMP pathway would therefore be beneficial.

We have previously demonstrated that short-term intravenous sodium nitrite improves cardiac and pulmonary haemodynamics in patients with HFrEF,^16^ and very recent studies have shown that nebulized nitrite improves rest and exercise haemodynamics in HFpEF and that sodium nitrite infusion improves exercise capacity in HFpEF.^17^^,^^18^ Although nitrite can be reduced to NO under hypoxic/acidic conditions some vasodilation is observed even under normoxic conditions, potentially via a NO-independent mechanism.^19^

We have therefore undertaken a study to evaluate the potential of nitrite to circumvent ‘platelet NO resistance’ and to compare responses to nitrite vs. the NO donor SNP in platelets from patients with HFpEF with chronic AF (HFpEF-AF) with those from healthy volunteers (HVs) and patients with chronic AF alone. Our results demonstrate that platelet NO resistance exists in HFpEF-AF patients, but is not observed in age-matched patients with chronic AF without HF. Since this phenomenon was observed in washed platelets; it must be largely independent of superoxide production by neutrophils and intrinsic to the platelet. We also show that platelet aggregation in HFpEF-AF is inhibited by nitrite, and that this effect was not impaired when compared with that seen in HV and chronic AF.

## 2. Methods

### 2.1 Subjects

We studied 12 HVs (8 males and 4 women), 29 patients diagnosed with HFpEF-AF (21 males and 8 women), 8 age-matched patients with chronic AF without HF or known coronary artery disease (4 males and 4 women). The HVs and patients were randomly assigned to the pharmacological experiments, with 8–11 participants per treatment group. Sixteen young HVs (9 males and 7 females) were also recruited for western blotting experiments. HVs were non-smokers free of any cardiovascular risk factors, not on any regular medications, and in particular had not taken anti-platelet drugs in the 10 days prior to the study. All patients met the established criteria for the diagnosis of HFpEF-AF.^20^^,^^21^ This included participants who have permanent AF and ejection fraction >55% (established by echocardiography during screening).^22^^,^^23^ Chronic AF participants included persistent AF with no evidence of HF or known coronary artery disease. All participants gave written consent before participation in the study. The investigation conforms to the principles outlined in the Declaration of Helsinki. The studies were approved by the University of Birmingham Ethics Review Committee (ERN_10-0625 and ERN_12-1184R2) and West Midlands Coventry and Warwickshire research ethics committee (14/WM/1211 and 12/WM/0344).

### 2.2 Blood sampling and platelet preparation

Venous blood was drawn in 9NC coagulation sodium citrate 3.2% vacutainer tube (Greiner Bio-One, Austria). A preparation of washed platelets was obtained as previously described in.^24^^,^^25^ Whole blood was centrifuged at 200 *g* for 20 min and platelet rich plasma (PRP) was collected up to 0.5 cm from the interface with the red blood cell (RBC) pellet in order to minimize RBCs contamination. Platelets were isolated from PRP by centrifugation at 1000 *g* for 10 min following addition of PGI_2_ (0.1 µg/ml; to inhibit platelet activation; Sigma Aldrich). The resulting platelet pellet was resuspended in Tyrode’s buffer (134 mM NaCl, 0.34 mM Na_2_HPO_4_, 2.9 mM KCl, 12 mM NaHCO_3_, 20 mM HEPES, 5 mM glucose, 1 mM MgCl_2_, pH 7.3) and centrifuged at 1000 *g* in the presence of 0.1 µg/ml PGI_2_. The supernatant was discarded and the platelet pellet was resuspended in Tyrode’s buffer. The washed platelet suspensions were allowed to rest for 1 h prior to experimentation to allow the effects of PGI_2_ to decay. The level of contamination of our washed platelet preparation with plasma constituents was determined using the Bio-Rad assay for protein determination.

### 2.3 Assessment of RBC and leukocyte contamination in washed platelet preparations

Flow cytometry was used to determine RBC and leukocyte contamination in washed platelet preparations. Platelets (2 × 10^8^/ml in Tyrode’s containing 10% heat deactivated human serum) and RBC (diluted 1:500 in Tyrode’s buffer) were stained with the RBC surface marker PE-conjugated anti CD235a (eBioscience) or isotype IgG control (eBioscience) for 20 min in the dark. Cells were washed and acquired with a C6 Accuri flow cytometer. RBC was gated using anti-CD235a (eBioscience) and applied to the washed platelet preparation to assess RBC contamination. To assess leukocyte contamination in preparations of washed platelets, platelets and leukocytes were labelled with anti-CD45 antibody-allophycocyanin (APC; Beckman Coulter) or isotype-APC control (Beckman Coulter) for 30 min at 4°C. In total 0.3 ml of blood was fixed using 2% formaldehyde for 10 min and after centrifugation at 500 *g* for 5 min, blood was resuspended in 3 ml ACK (Ammonium-Chloride-Potassium) lysis buffer for 10 min to remove RBCs. Cells were labelled and washed in PBS at 500 *g* for 5 min and resuspended in 300 μl PBS for analysis on a C6 Accuri flow cytometer. Leukocyte populations isolated from whole blood were separated based on forward scatter and CD45 expression. Gates based on this distribution were used to assess leukocytes present in preparations of washed platelets.

### 2.4 Oxyhaemoglobin preparation

Human haemoglobin (Sigma Aldrich) was dissolved in water (20 mg/ml) and reduced by a 10-fold molar excess of sodium dithionite (Na_2_S_2_O_4_; Sigma Aldrich). Excess reductant was removed by gel filtration over Sephadex G-25 (PD10 desalting column; GE Healthcare) according to the manufacturer’s instructions. Oxyhaemoglobin (OxyHb) was eluted with 3.5 ml of water, and only the middle run was collected. The concentration of OxyHb was determined spectrophotometrically, as described in.^26^ Aliquots of the OxyHb stock solution were kept at −80°C, thawed on the day of experimentation and discarded after use.

### 2.5 Platelet aggregation

Washed platelets were suspended at 2 × 10^8^/ml for light transmission aggregation using a lumi-dual aggregometer (model 460VS; Chronolog, Labmedics) under continuous stirring at 1200 rpm, as previously described in.^24^ Sodium nitrite, sodium nitrate, sodium nitroprusside (SNP), 1H-[1, 2, 4]Oxadiazolo[4, 3-a]quinoxalin-1-one (ODQ), 2-Phenyl-4, 4, 5, 5-tetramethylimidazoline-1-oxyl 3-oxide (PTIO) (all purchased from Sigma Aldrich), OxyHb, BAY 41-2272, or vehicles were incubated for the stated time and concentrations as indicated in the figure legends before platelet activation with 3 µg/ml collagen (Nycomed).

### 2.6 Western blotting

Washed platelets were suspended at 5 × 10^8^/ml for western blot experiments. Platelets were incubated as indicated in the figure legends. Incubations were stopped by adding 5× reducing sample buffer at the indicated time point. Samples were boiled for 5 min and spun prior to sodium dodecyl sulphate-polyacrylamide gel electrophoresis (SDS-PAGE) (10%) and transferred onto a polyvinylidene fluoride (PVDF; GE Healthcare) membrane. PVDF membranes were incubated with antibodies against p-Serine 239 VASP (Cell signalling Technology) and α-tubulin (Sigma Aldrich) overnight at 4°C. Membranes were then incubated with appropriate secondary antibodies [anti-rabbit (GE healthcare) and anti-mouse (Dako)] as detailed in figure legend text. Band densitometry was performed as previously described in.^27^

### 2.7 Statistical analysis

Results are presented either as representative experiment of n experiments performed or as average ± SEM. Differences between groups were analysed using the, Fisher’s exact test, unpaired *t* test, one- or two-way ANOVA by Dunnett’s or Sidak’s multiple comparisons test as appropriate. Within group differences was assessed by Wilcoxon matched-pairs signed rank test. *P* < 0.05 was considered significant. All analyses were performed using Prism version 7.0 (GraphPad Inc., Lo Jolla, CA).

## 3. Results

### 3.1 HVs and HF patients’ characteristics

*Table* *1* summarizes the subject characteristics including drug therapies. Patients and healthy controls were well matched in terms of age (*P* = 0.40; one-way ANOVA) and gender (*P* = 0.53; Fisher’s exact test). Young HVs (whose blood was used for platelet isolation and western blotting) were well matched in terms of gender to HFpEF-AF group (*P* = 0.33; Fisher’s exact test).

**Table 1:** Subject characteristics of HVs, HFpEF with chronic AF, and chornic AF

Parameter	HVs (*n* = 12)	HFpEF with chronic AF (*n* = 29)	Chronic AF (*n* = 8)
Sex (M/F)	8/4	21/8	4/4
Age, year (mean ± SD)	71.3 ± 5.9	74.3 ± 6.2	73.9 ± 7.9
NYHA-Class I/II/III/	–	23/11/2	–
Diabetes mellitus	–	2	2
ACE-inhibitors	–	10	2
Angiotensin II receptor blocker	–	2	2
Diuretics	–	2	4
β-adrenoceptor antagonists	–	8	6
α-adrenoceptor antagonist	–	2	–
Calcium channel blocker	–	9	1
Statins	–	10	2
Cardiac glycoside	–	–	–
Digoxin	–	4	2
Anti-arrhythmic	–	–	1
Anti-diabetic	–	2	2
Anti-coagulant	–	26	8

Values are mean ± SD.

NYHA, New York Heart Association classification; HFpEF, Heart Failure with preserved ejection fraction; ACE-inhibitors, angiotensin-converting enzyme inhibitors.

### 3.2 Washed platelet preparation

**Figure 1:**
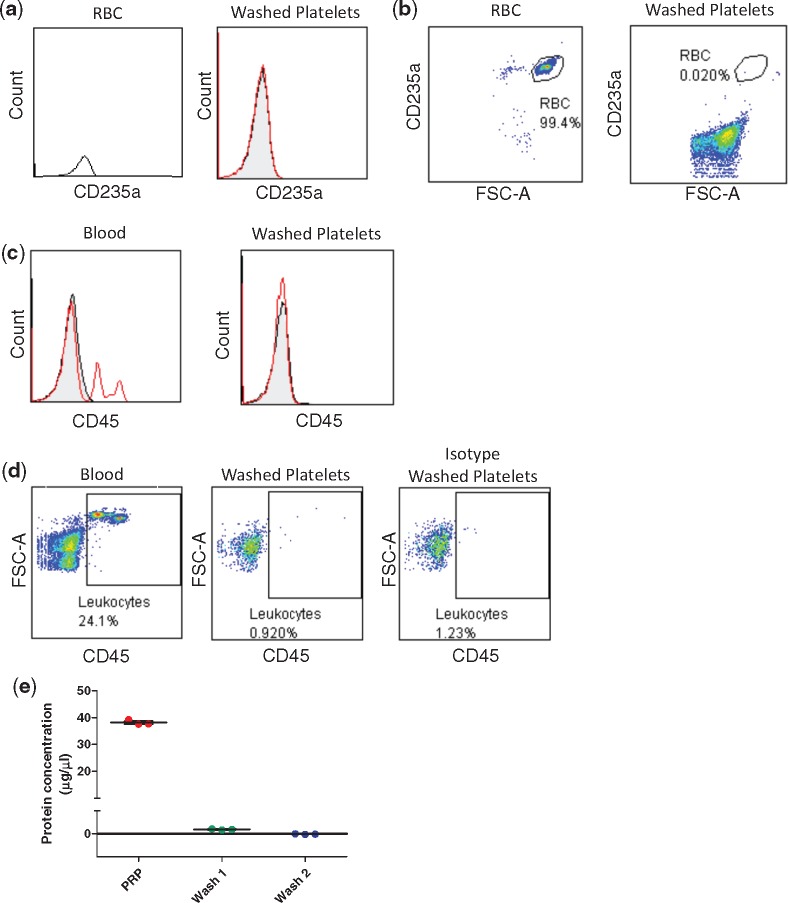
Staining of RBCs and leukocytes in a preparation of washed platelets. RBCs and platelets were stained with 0.2 µg anti-CD235a PE-conjugated antibody or with IgG isotype control. Representative overlay plots are shown (RBCs on the left and platelets on the right) (*A*). A gate was drawn to include 97–99% of RBCs and applied to the washed platelet sample; cells in the gate were counted as RBCs contaminating the preparation (*B*, *n* = 8). RBCs were lysed in whole blood and the remaining cells or a suspension of washed platelets were stained with APC-conjugated anti-CD45 or with IgG isotype control. Representative overlay plots are shown (blood on the left and platelets on the right) (*C*) A gate was drawn around the leukocyte population (*D*, left panel) and applied to the washed platelet sample stained with CD45 or isotype control (*D*, centre and right panel, respectively) (*n* = 3). (*E*) Assessment of plasma protein contamination was determined in washed platelets (first and second wash) and compared with PRP (*n* = 3).

All experiments presented in this study utilized a preparation of washed platelets in order to exclude the interference of other blood cell types and to minimise the contribution of plasma proteins and other blood cell types to the effects that nitrite may have on platelet function. RBCs in particular contain (haeme-mediated) nitrite reductase activities, which are able to reduce nitrite to NO, potentially augmenting the inhibitory effects of nitrite on platelets. Perhaps even more importantly, RBCs also contain high concentrations of oxygenated haemoglobin, which is an effective NO scavenger. Contamination of RBCs was evaluated in our platelet preparation by FACS as shown in *Figure 1A *and* B*, <0.035 ± 0.02% RBC contamination was present in a preparation of washed platelets. Contamination by leukocytes was also excluded using a similar approach. *Figure 1C *and* D* shows that CD45 positive cells were effectively absent from washed platelet preparations; on average fewer than 1 leukocyte per 1000 platelets were found to be present (*Figure 1D*), and plasma proteins concentration was confirmed to be negligible in the second washed platelets (−2 ± 0.01%) when compared with PRP (100 ± 0.55%) and first wash (20 ± 0.01%; *Figure 1E*).

### 3.3 Nitrite circumvents ‘platelet NO resistance’ in patients with HFpEF and chronic AF

**Figure 2:**
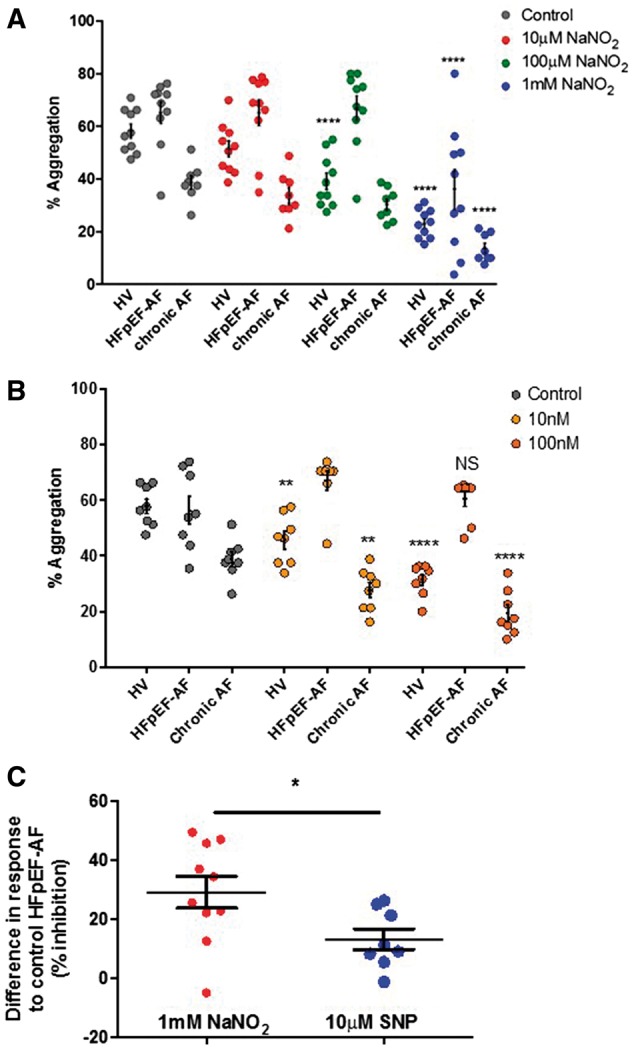
Platelet aggregation is inhibited by NaNO_2_. 2 × 10^8^/ml washed platelets from HVs, HFpEF-AF and chronic AF alone were incubated with increasing concentrations of NaNO_2_ or SNP for 5 min, activated with 3 µg/ml collagen (*A* and *B*) and studied by light transmission aggregometry. Statistical differences were determined by repeated measures two-way ANOVA with Dunnett’s test for multiple comparisons with the control dose values (**P < 0.01, ****P < 0.0001; *n* = 8–10). (*C*) Platelet responses to nitrite and SNP in the HFpEF-AF group. The difference between nitrite (1 mM) and SNP (10 µM) responses to collagen with their respective control HFpEF were analysed. *P < 0.05 unpaired *t* test with Welch’s correction (*n* = 8–10).

Concentration response to collagen (1, 3, and 10 μg/ml) was conducted to determine the concentration used for platelet aggregation experiments (data not shown). A collagen dose (3 μg/ml) that achieved a 50% aggregatory response in platelets was selected for this study.^24^ The effects of nitrite on platelet aggregation in HV, HFpEF-AF, and chronic AF patients alone were studied using 3 μg/ml collagen (*Figure 2A*). Incubation with nitrite in HVs induced significant concentration-dependent inhibition of platelet aggregation in response to collagen (*P* < 0.0001 at 100 µM and 1 mM; *n* = 10; *Figure 2A*), indicating that nitrite is able to affect platelet function in the absence of other cell types or extracellular proteins. A similar trend was also observed with the NO donor, SNP, with significant concentration-dependent attenuation of platelet aggregation at 10 and 100 nM in response to collagen (*P* < 0.01 at 10 nM and *P* < 0.0001 at 100 nM; *n* = 8; *Figure 2B*).

We next investigated whether nitrite inhibited platelet aggregation following administration of 3 μg/ml collagen (*Figure 2A*) in patients from HFpEF-AF. Nitrite significantly attenuated platelet aggregation with 1 mM nitrite (*P* < 0.0001; *n* = 10), whilst SNP (10 and 100 nM) showed no diminution of response to collagen activated platelets (*n* = 8; *Figure 2B*). To determine whether increased concentrations (1 and 10 µM) of SNP attenuated platelet aggregation in HFpEF-AF group, we observed that SNP caused 45.20 ± 5.45 and 43.11 ± 5.0% inhibition in aggregation, respectively (see Supplementary material online, *Figure S1*).

To validate whether the SNP responses to collagen were more markedly affected than those of nitrite in the HFpEF-AF group, we compared the differences in nitrite and SNP response to collagen at the highest doses used to their respective control HFpEF-AF. As shown in *Figure 2C*, 10 µM SNP (13.15 ± 3.5% inhibition) demonstrated impaired responses compared with 1 mM nitrite (29.12 ± 5.4% inhibition; *P* < 0.05).

**Figure 3:**
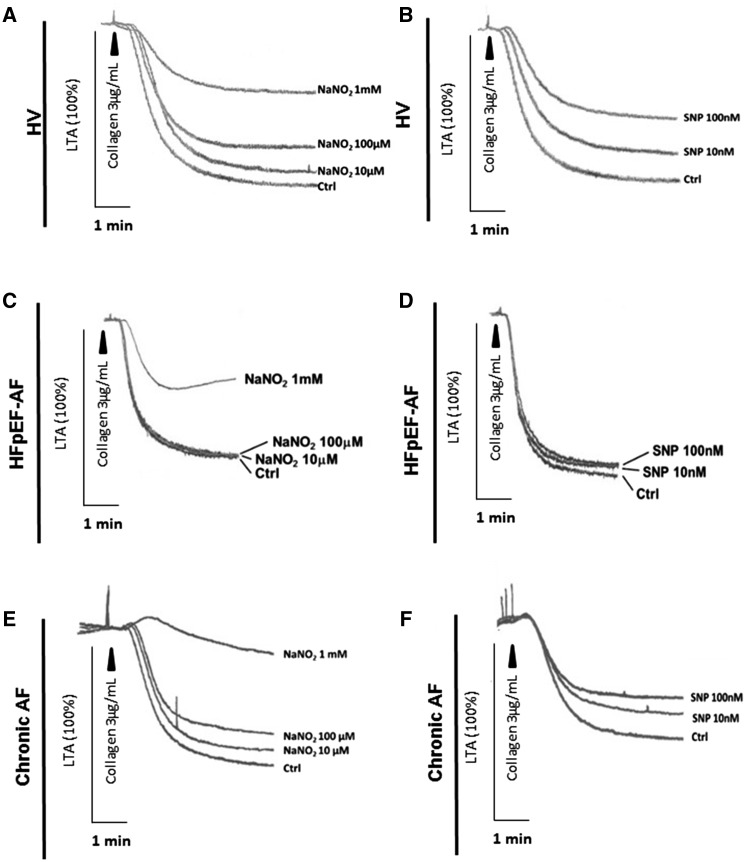
Representative platelet aggregation traces for experiments performed from *Figure 2* are shown. Platelet responses to nitrite and SNP from HVs (*A* and *B*, HV), HF with preserved ejection fraction with chronic AF (*C* and *D*, HFpEF-AF) and chronic AF (*E* and *F*, chronic AF).

To ascertain whether the platelet responses observed with nitrite and SNP in HFpEF-AF patients were attributable to the HF, the chronic AF or both we then measured platelet aggregation responses in a sub-group of patients with chronic AF alone. Both nitrite and SNP induced concentration-dependent attenuation of platelet aggregation in response to collagen (*Figure 2A*; *P* < 0.0001 nitrite (1 mM); and *Figure 2B*; *P* < 0.01 (10 nM) and *P* < 0.0001 (100nM) SNP, respectively). Representative traces for HV, HFpEF-AF and cAF alone are shown in *Figure 3*.

**Figure 4:**
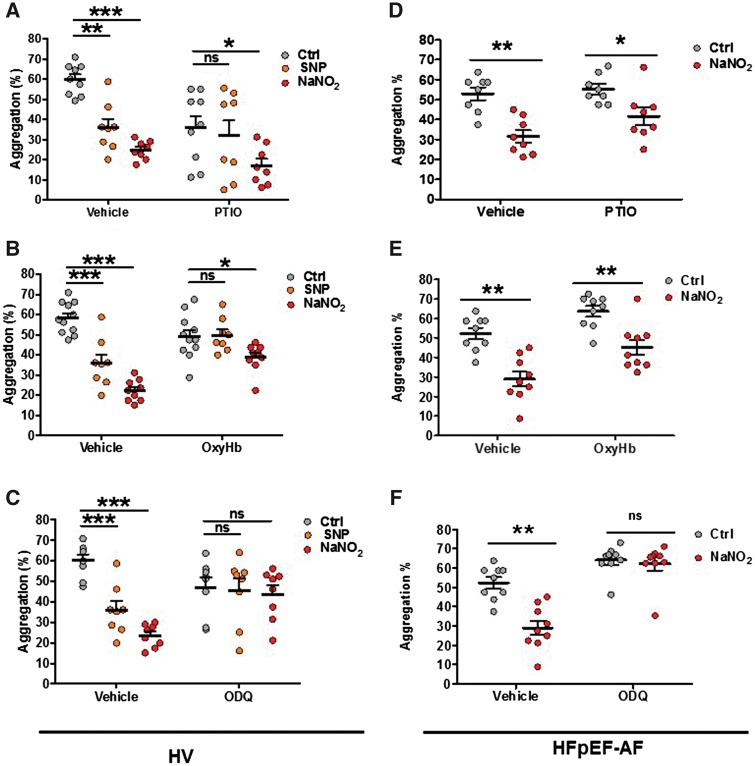
NaNO_2_ effects on aggregation depend on sGC. 2 × 10^8^/ml washed platelets from HVs (*n* = 8–11) were incubated with NO scavengers (*A*) 100 µM PTIO or (*B*) 10 µM OxyHb immediately before addition of 1 mM NaNO_2_ or 100 nM SNP, or (*C*) sGC inhibitor 10 µM ODQ 15 min. 2 × 10^8^/ml washed platelets from HFpEF-AF patients (*n* = 8–9) were incubated with (*D*) PTIO, or (*E*) OxyHB or (*F*) ODQ. Aggregation was triggered by 3 µg/ml collagen. Representative traces for all the performed experiments are shown in Supplementary material online, *Figure S2*. Repeated measures one-way ANOVA with Dunnett’s test for multiple comparisons was performed to compare SNP and nitrite to control. Differences between control scavengers/inhibitors to nitrite was evaluated by Wilcoxon matched-pairs signed rank test (*P < 0.05, **P < 0.01, ***P < 0.001).

To assess the mechanism by which high concentrations of nitrite circumvent platelet NO resistance, we first assessed whether nitrite is converted to NO under these experimental conditions by using two different NO scavengers (PTIO and OxyHb). In both HVs [*Figure 4A* (*n* = 8) and *Figure 4B* (*n* = 11)] and patients with HFpEF [*Figure 4D* (*n* = 8) and *Figure 4E* (*n* = 9)], neither PTIO nor OxyHb were able to revert the inhibition caused by a high concentration of nitrite on platelet aggregation. In order to test the efficacy of PTIO and OxyHb we also assessed aggregation in HVs in the presence of the NO donor SNP. As depicted in *Figure 4A *and* B*, inhibition to SNP was reversed by both NO scavengers.

To delineate the possible role for sGC, we used the sGC inhibitor, ODQ in combination with an inhibitory concentration of nitrite and studied collagen aggregation (*Figure 4C *and* F*). ODQ reverted SNP-dependent inhibition in HV and nitrite-dependent inhibition in both healthy subjects (*Figure 4C*; *n* = 8) and HFpEF-AF (*Figure 4F*; *n* = 9), indicating that nitrite indeed acts through sGC. Representative traces for *Figure 4* are shown in Supplementary material online, *Figure S2*. Interestingly, the sGC dependent effect of nitrite on HV platelets exhibited synergistic activity with the NO-independent sGC activator, Bay 41-2272 (*P* < 0.001 30 nM Bay 41-2272 plus 10 and 100 µM nitrite, respectively; *n* = 11, see Supplementary material online, *Figure S3B*). A similar trend was also observed with SNP as a positive control (30 nM Bay 41-2272 plus 10 nM SNP (*P* < 0.01) and 100 nM SNP (*P* < 0.001), respectively; see Supplementary material online, *Figure S3C*).

### 3.4 Nitrite phosphorylates serine-239 VASP

**Figure 5:**
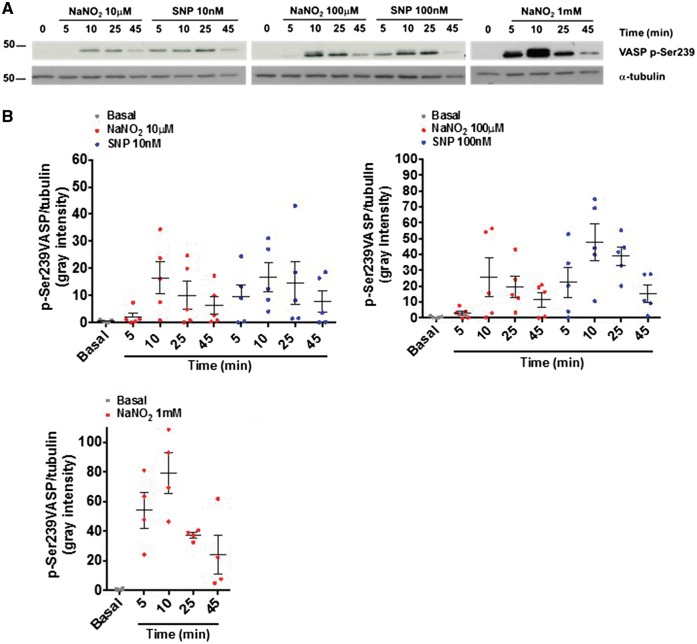
NaNO_2_ triggers phosphorylation of VASP. 5 × 10^8^/ml washed platelets from healthy young volunteers were incubated with increasing concentrations of NaNO_2_ or SNP and lysed by addition of SDS containing 5× sample buffer at the indicated time point. Aliquots of the lysates were used for SDS-PAGE (10%). Blots were probed with anti p-VASP Serine 239 (1:1000) or α-tubulin (1:5000) and the appropriate secondary antibodies (anti-rabbit or anti-mouse 1:10000). Representative blots (*A*) and densitometrical analysis of all the experiments performed (*B*, *n* = 4–5) are shown.

To confirm that the effects of nitrite on platelet aggregation occur via the activation of the sGC-cGMP pathway, we next studied the status of phosphorylation of the cyclic nucleotide downstream substrate VASP. In order to understand the normal physiology vs disease physiology of nitrite mechanism in washed platelets, VASP serine-239 phosphorylation (mainly dependent on cGMP elevation) was assessed from young HVs vs. HFpEF-AF (*Figure 5A *and* B*). VASP serine-239 phosphorylation was increased with nitrite in a concentration-dependent manner as compared with the NO donor SNP. All concentrations of nitrite reached a peak after 5–10 min of incubation and then decreased to low levels at 45 min. VASP serine-239 phosphorylation with 100 µM and 1 mM nitrite were similar to that triggered by treatment with 10 and 100 nM SNP, respectively (*n* = 5).

**Figure 6:**
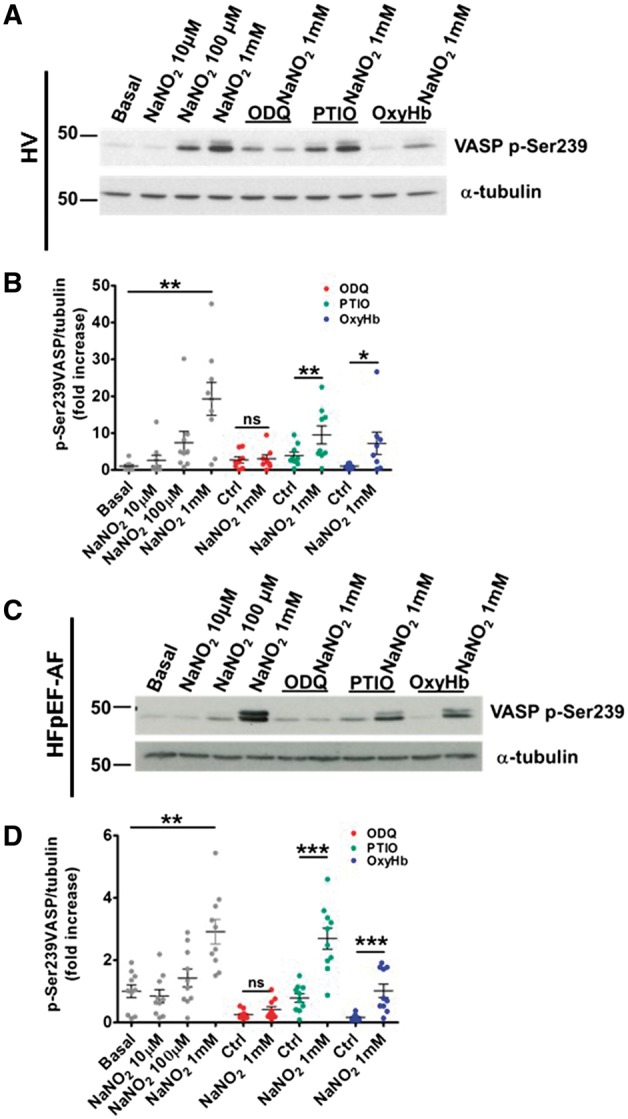
NaNO_2_ upregulates VASP serine-239 phosphorylation NO-independently. 5 × 10^8^/ml washed platelets from HVs (*A* and *B*; *n* = 8–9) or HFpEF-AF patients (*C* and *D*; *n* = 10) were incubated with ODQ, PTIO, or OxyHb before addition of NaNO_2_ for 10 min and lysed by addition of 5× sample buffer. Aliquots of the lysates were used for SDS-PAGE (10%) and western blot as in *Figure 5*. Representative blots (*A* and *C*) and densitometrical analysis of all the experiments performed (*B* and *D*) are shown. Statistical differences were determined by one-way ANOVA and Dunnet’s post-test. Differences within groups (control NO scavengers/sGC inhibitors vs. nitrite) were evaluated by Wilcoxon matched-pairs signed rank test (*P < 0.05; **P < 0.01; ***P < 0.001).

To determine whether the underlying mechanism of nitrite was NO dependent or independent, we measured serine-239 phosphorylation of VASP following the use of PTIO and OxyHb. We found that in the presence of NO scavengers, VASP serine-239 was phosphorylated by nitrite in both young HVs (*Figure 6A *and* B*; *n* = 9) and HFpEF-AF patients (*Figure 6C *and* D*; *n* = 10). We next investigated whether nitrite activated sGC to phosphorylate serine-239 VASP. VASP phosphorylation on Serine-239 triggered by nitrite was completely blocked by ODQ in both HVs and patients with HFpEF-AF, thus corroborating our findings from platelet aggregation experiments (*Figure 4A–F*).

### 3.5 Nitrate does not affect platelet aggregation and VASP phosphorylation

**Figure 7:**
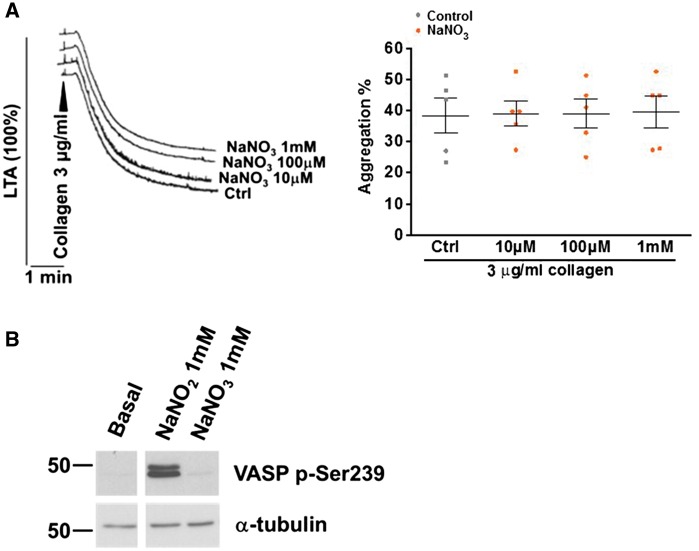
NaNO_3_ has no inhibitory effects in washed platelets 5 × 10^8^ /ml washed platelets from HVs were incubated with increasing concentrations of NaNO_3_ (*A*, *n* = 5) for 5 min, activated with 3 µg/ml collagen. 5 × 10^8^/ml washed platelets from healthy subjects were incubated with 1 mM NaNO_3_, lysed after 10 min and used for SDS-PAGE and western blot as above. A representative blot is shown (*B*, *n* = 4).

The effects of oral inorganic nitrate are usually considered to be dependent on enterosalivary circulation of nitrate with reduction of salivary nitrate to nitrite by oral bacteria, but a direct effect of nitrate cannot be excluded. We therefore assessed the effect of a high concentration of sodium nitrate (1 mM) added *in vitro* on collagen-induced aggregation in young HVs (*Figure 7A*). In the absence of a functional nitrate reductase, the addition of nitrate to platelets *in vitro* had no effect on aggregation (*P* > 0.05; *n* = 5) or phosphorylation of VASP serine-239 (*Figure 7B*).

## 4. Discussion

In this study, we show several important new findings: first, platelet NO resistance exist in patients with HFpEF with associated chronic AF compared with age-matched HVs. Second, while NO resistance has been observed in recent onset AF^7^ we did not observe it in age-matched patients with chronic AF in the absence of HF. Taken together, these data suggest that the platelet NO resistance is attributable predominantly to the HFpEF rather than the associated chronic AF. Third, this NO resistance was observed in washed platelets indicating that platelet NO resistance is largely intrinsic to the platelet. Fourth, nitrite inhibits platelet aggregation in HFpEF-AF patients by circumventing the platelet NO resistance phenomenon by directly activating sGC and phosphorylating VASP serine 239 independent of NO and the interaction with other formed elements of blood and their constiuents such as mitochondria (neutrophils) or haemoglobin (RBCs).

Approximately half of all cases of HF have HFpEF and the syndrome is frequently associated with AF.^28^ The pathophysiology of HFpEF is associated with abnormalities in left ventricular diastolic reserve, impaired systolic reserve, peripheral and pulmonary vasodilatation, endothelial dysfunction, and right ventricular dysfunction related to the presence of pulmonary hypertension (PH).^29^

Recent studies have postulated that impairment of the NO-cGMP pathway may also play a contributing factor in the haemodynamic abnormalities observed in HFpEF.^30^^,^^31^ However, to our knowledge the role of the NO-cGMP pathway on platelet function in patients with HFpEF with chronic AF has not been previously evaluated. It is well-documented that impaired platelet responsiveness to NO donors (organic nitrates and SNP) occurs in a number of cardiovascular disease states, such as ischaemic heart disease, HFrEF, and in new onset of AF.^4^^,^^7^^,^^15^ Herein, we sought to determine whether NO donors also have a similar impact on the function of platelets isolated from HFpEF patients with chronic AF. We demonstrate for the first time that ‘platelet NO resistance’ exists in HFpEF associated with chronic AF. We used SNP, a well-established NO donor, to evaluate the impact of activation of the sGC-cGMP pathway on platelet aggregation.^4^^,^^32^ Platelet responsiveness to the NO donor SNP from HFpEF-AF were diminished following platelet activation with collagen when compared with platelets from HVs.

It is now appreciated that under certain conditions nitrite can serve as an alternative source of NO in the vasculature and other tissues.^19^^,^^33^ Recent studies have shown that the nitrate-nitrite-NO pathway inhibits platelet activation.^34–36^ It has also been suggested that platelets possess the ability to generate transient NO signals from nitrite in the absence of other blood cell types through an unidentified mechanism.^34^

Although the role of endothelial-derived NO on platelet function is well characterized,^37^ the mechanism(s) by which nitrite inhibit platelet aggregation remain poorly defined. Platelets are exposed not only to endothelial NO but also to circulatory nitrite, and this could represent a source of NO and an alternative mechanism of control of platelet activation. Basal circulating nitrite levels in human plasma range are typically in the submicromolar range^38^^,^^39^ and this can rise to levels >8 µM upon therapeutic nitrite application.^17^^,^^18^ However, local concentrations in the microcirculation of mucosal membranes are likely considerably higher. In the oral cavity, nitrite concentrations reach micromolar concentrations even under normal physiological conditions, and salivary nitrite concentrations may reach 1–2 mM after dietary nitrate.^40^^,^^41^ If nitrite were to be used as a therapeutic agent to treat HFpEF-AF, concentrations of this mediator would exceed those physiological levels by far, with possible effects on platelet function. A similar scenario is likely to occur at the alveolar-arterial interface of the pulmonary circulation following therapeutic application of nebulized nitrite.

This study was motivated by the paucity of information on the signalling cascade leading to platelet inhibition by nitrite. Although it is generally assumed that nitrite requires prior reduction to NO to become bioactive, this process is remarkably inefficient under normoxic conditions, and not all of its actions appear to be accompanied by the generation of free NO.^19^^,^^42^ To determine whether nitrite inhibits platelet aggregation independently of haemoglobin, we used a well-established washed platelet aggregation technique.^43^ Washed platelets are routinely used to assess pharmacological agents on platelet function and mechanism in the absence of plasma proteins, enzymes, and blood cells. Our results demonstrate that platelets from HV are inhibited by nitrite through the activation of sGC (as assessed via the sGC inhibitor ODQ; *Figure 4*). We next sought to determine whether these effects were NO dependent. Both NO scavengers (PTIO and OxyHb) effectively reverted the effects of an inhibitory concentration of SNP on platelet aggregation (*Figure 4*). In contrast neither PTIO nor OxyHb were able to revert the inhibition caused by a high concentration of nitrite on platelet aggregation from HV. VASP phosphorylation on Ser239 triggered by nitrite was completely blocked by ODQ (*Figure 6*), but inhibition was only partial with PTIO and OxyHb (*Figure 6*). Collectively, these data indicate that the antiaggregatory effects of nitrite are only partly mediated by NO. This is consistent with additional findings showing that the effects of nitrite are potentiated by the NO-independent sGC activator Bay 41-2272 (see Supplementary material online, *Figure S3*). It is conceivable, therefore, that part of the effects of nitrite are mediated by a modulation of (one or more) cysteine thiols of sGC; whether this is via S-nitrosation or S-thiolation (e.g. sulphydration or formation of a mixed disulphide with another low-molecular weight thiol such as cysteine or glutathione, and therefore oxidation of a regulatory thiol in sGC)^13^^,^^14^ or another mechanism (such as the loss of haeme, which renders sGC unresponsive to NO) was beyond the remit of the present study and warrants further investigation.

Previous studies have suggested that nitrite therapy may not be subject to the development of tolerance.^44^ Furthermore, we and others have very recently explored nitrite as a potential treatment of HF. We have shown that short-term intravenous sodium nitrite improves cardiac and pulmonary in patients with HFrEF,^16^ and very recent studies by Borlaug *et al.*^18^ have demonstrated that acute nitrite infusion or nebulized nitrite improves rest and exercise haemodynamics and exercise capacity in HFpEF.^17^ To assess whether nitrite inhibits platelet aggregation in this group of patients, we next assessed the effects of nitrite on platelet function. In the presence of ‘NO resistance’, nitrite inhibits platelet aggregation. To assess the potential mechanism by which nitrite mediates these promising effects, platelet responses to nitrite was assessed in the presence of sGC inhibitor or NO scavengers. We show that the platelet responsiveness to nitrite was significantly attenuated in the presence of ODQ (*Figure 4*), but both NO scavengers were unable to revert the inhibition caused by a high concentration of nitrite on platelet aggregation and VASP serine 239 phosphorylation (*Figure 6*). Herein, we show for the first time that nitrite circumvents the platelet NO resistance in patients with HFpEF-AF. A similar result was also observed with our western blot data. However, haeme-containing components (cytochrome C) of the mitochondrial respiratory chain of platelets may convert nitrite to NO^45^ inside of the platelet, thus avoiding the scavenging by OxyHb or PTIO.

A number of studies have associated AF with inflammation, endothelial dysfunction and in particular in the new onset AF with impaired platelet NO response.^7^ Procter *et al.* (2015) have previously reported that acute onset AF is associated with platelet NO resistance, whilst responses to NO donor (SNP) were not impaired in chronic AF patients. To validate whether the platelet NO resistance observed in the HFpEF-AF with SNP was HFpEF dependent and not AF, we also assessed the effects of nitrite and SNP in a sub-group of patients with chronic AF alone. There was approximately two-fold variability between HFpEF-AF vs chronic AF subgroups with SNP, therefore further supporting platelet NO resistance in patients with HFpEF-AF. In addition, nitrite also inhibited platelet aggregation in a dose-dependent manner in the chronic-AF sub-group.

ACE inhibitors have previously been shown to reduce platelet NO resistance.^46^ Although 10 of the 29 patients with HFpEF-AF were taking ACE inhibitors, NO resistance was observed whether or not the patients were taking ACE inhibitors. Two of the eight patients with chronic AF alone were taking ACE inhibitors, but in contrast platelet NO responsiveness was not observed in either of these two patients or the six who were not taking ACE inhibitors.

Previous studies have demonstrated that cGMP plays a key role in determining the functional and haemodynamic abnormalities developing in HFpEF.^30^ In this study, we show that cAF in the absence of HF respond to NO donors and there is a clear difference in response of ‘NO resistance’ in patients with HFpEF-AF. This suggests that phenotyping of patients according to their ‘NO-stimulatable’ component in platelet reactivity might allow to stratify them to different pharmacological treatment (e.g. personalised medicine).

Herein, we have compared the effects of nitrite on platelet function in patients with chronic AF with and without HFpEF to conclude about HFpEF. This study could have benefited in having an additional sub-group of HFpEF patients with sinus rhythm to further validate the study. However, as local practice, the majority of HFpEF in sinus rhythm were taking aspirin and therefore we were unable to recruit for this study. Thus, this remains a major limitation to the current investigation and further studies are warranted to clarify the role of nitrite on platelet function in this particular patient cohort.

A major limitation of the current findings is that the concentrations of nitrite used in this study are clearly pharmacological, therefore the observations do not imply a physiological role for normal plasma nitrite concentrations in the modulation of platelet function. Nevertheless, they may have implications for therapy. Recent studies have suggested a potentially beneficial effect of the nitrate/nitrite pathway on haemodynamics and exercise capacity in HFpEF, this study raises the potential that such therapy may also reduce the burden of thromboembolic disease seen HFpEF patients with chronic AF.

Furthermore, we observed a decreased platelet aggregation response to collagen in the control chronic AF when compared with age-matched control groups from HVs and HFpEF-AF patients. To our knowledge the effects of collagen on washed platelet aggregation from chronic AF has not been investigated before, but we have previously assessed platelet aggregation (PRP) in patients with AF on no therapy to have 24% inhibition of aggregation in response to 2 mg/ml collagen.^47^ We have reported the complexity of the events of platelet activation in AF and therefore further studies are warranted to clarify the role of nitrite and NO donors in the disease states investigated herein.

In conclusion, we show for the first time that HFpEF is associated with marked impairment of platelet NO responses and that nitrite circumvents the ‘platelet NO resistance’ phenomenon in human HFpEF, at least in part by acting as a sGC activator. Our findings are consistent with the notion that thiol oxidation at the level of the NO-receptor, sGC may play a major role in the phenomenon of ‘NO resistance’; this would seem to warrant further investigation. Our findings may also have relevance to the condition of PH since coagulation has been implicated in the pathogenesis of this disease. Although the use of anticoagulation as part of the treatment in PH remains controversial, recent experimental studies suggest that nitrite may provide some benefit in this setting.^48^ Thus, our data implicate an alternative strategy to potentiate efficacy of direct sGC activation, and perhaps may reduce the risk of thrombotic complications in PH. 

## Supplementary material

Supplementary material is available at *Cardiovascular Research* online.

## Supplementary Material

Supplementary Data
