# Supplementary material for: Nitrite circumvents platelet resistance to nitric oxide in patients with heart failure preserved ejection fraction and chronic atrial fibrillation
Source: Cardiovasc Res. 2018 Apr 12;114(10):1313–23. doi: 10.1093/cvr/cvy087 (PMC6054254; doi:10.1093/cvr/cvy087)
Supplement: Supplementary Data [file cvy087_supp.zip › cvy087-suppl_data/supplementary material Feb2018_Final.docx]

**Supplementary Material**

**Nitrite circumvents platelet resistance to nitric oxide in patients with heart failure preserved ejection fraction**

Alessandra Borgognone^1, 3^, Eduard Shantsila^2^, Sophie M Worrall^1^, Eakkapote Prompunt^1^, Thomas Loka^1^, Brodie Loudon^4^, Myriam Chimen^1^, G Ed Rainger^1^, Janet Lord^3^, Ashely Turner^2^, Peter Nightingale^4^, Adrian J Hobbs^5^, Martin Feelisch^3^, Paulus Kirchoff^1^, Gregory Y Lip^2^, Steve P Watson^1^, Michael P Frenneaux^4*^and Melanie Madhani^1*^.

^1^Institute of Cardiovascular Sciences, University of Birmingham, Edgbaston, Birmingham, B15 2TT, UK

^2^Institute of Cardiovascular Sciences, University of Birmingham, City Hospital, Dudley Road, Birmingham, B18 7QH, UK

^3^Institute of Inflammation and Ageing, University of Birmingham, Edgbaston, Birmingham, B15 2TT, UK

^4^Wellcome Trust Clinical Research Facility, Queen Elizabeth Hospital, Edgbaston, Birmingham, B15 2TT, UK

^5^William Harvey Research Institute, Barts and The London School of Medicine, Queen Mary University of London, London, UK.

^6^Clinical and Experimental Sciences, Faculty of Medicine, University of Southampton, Southampton, UK

^7^ Norwich Medical School, University of East Anglia, Norwich, UK

**Supplementary Material**

**Figure 1. The effect of increasing concentration of SNP on platelet aggregation.** 2x10^8^/ml washed platelets from HFpEF with chronic were incubated with increasing concentrations of SNP for 5 min, activated with 3µg/ml collagen and studied by light transmission aggregometry (LTA). Statistical differences were determined by repeated measures two-way ~~one-way~~ ANOVA with Dunnett’s test for multiple comparisons (*p<0.05) n= 8

**Figure 2.** Representative platelet aggregation traces for experiments performed from Figure 4 are shown. Platelet responses to nitrite and SNP from healthy volunteers incubated with (a) PTIO, (b) OxyHb, and (c) ODQ. Platelet responses to nitrite in heart failure patients with chronic atrial fibrillation incubated with (d) PTIO, (e) OxyHb, and (f) ODQ.

**Figure 3. ~~Figure 2~~. NaNO_2_ effects on aggregation depend on sGC.** 2x10^8^/ml washed platelets from age-matched healthy volunteers (n= 3) were incubated with (a) sGC activator Bay 41-2272 (10-100nM) for 3 min before the addition of 3µg/ml collagen. Platelet aggregation was monitored for 5 min. Repeated measures one-way ANOVA with Dunnett’s test for multiple comparisons was performed to compare Bay 41-2272 to control (*p<0.05; **p<0.01; ***p<0.001). In some experiments, 2x10^8^/ml washed platelets were incubated with increasing concentrations of (b) NaNO_2_ or (c) SNP for 5 min before the addition of collagen 3µg/ml collagen. Bay 41-2272 (30nM) was added 1 min before the addition of collagen (n=11). Differences between Bay 41-2272 to nitrite (***p<0.0003) and SNP (**p<0.001; ***p<0.0003) was evaluated by repeated measures two-way ANOVA followed by Sidak’s multiple comparisons test.
