# Supplementary figures and images for: Nitrite circumvents platelet resistance to nitric oxide in patients with heart failure preserved ejection fraction and chronic atrial fibrillation
Source: Cardiovasc Res. 2018 Apr 12;114(10):1313–23. doi: 10.1093/cvr/cvy087 (PMC6054254; doi:10.1093/cvr/cvy087)

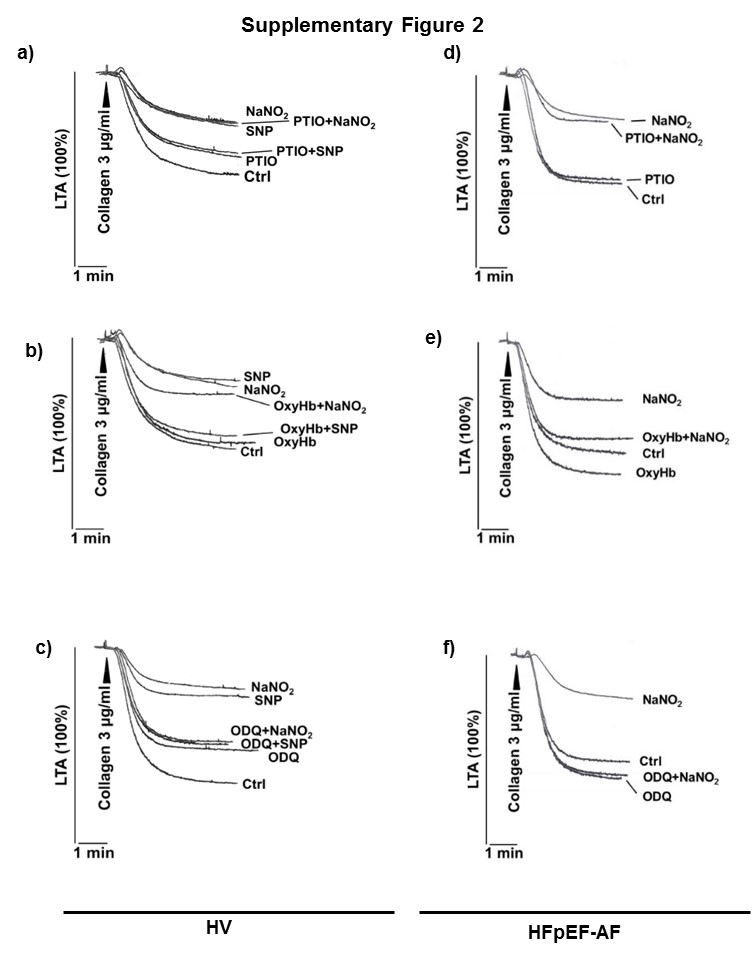

Supplement: Supplementary Data [file cvy087_supp.zip › cvy087-suppl_data/Supplementary Figure 2.jpg]

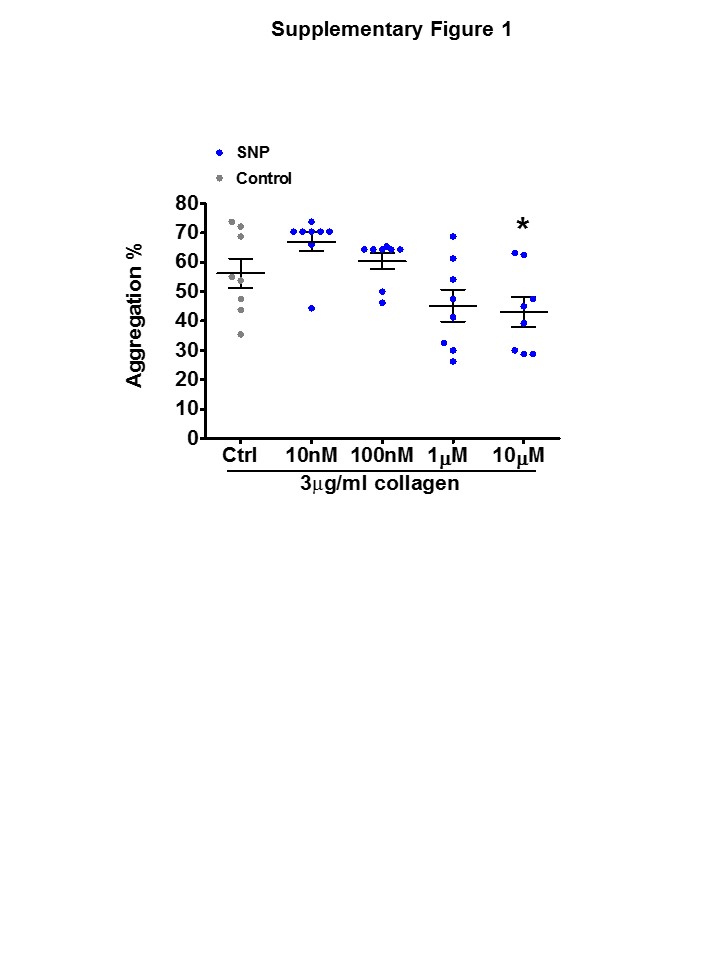

Supplement: Supplementary Data [file cvy087_supp.zip › cvy087-suppl_data/Supplementary Figure_1.jpg]

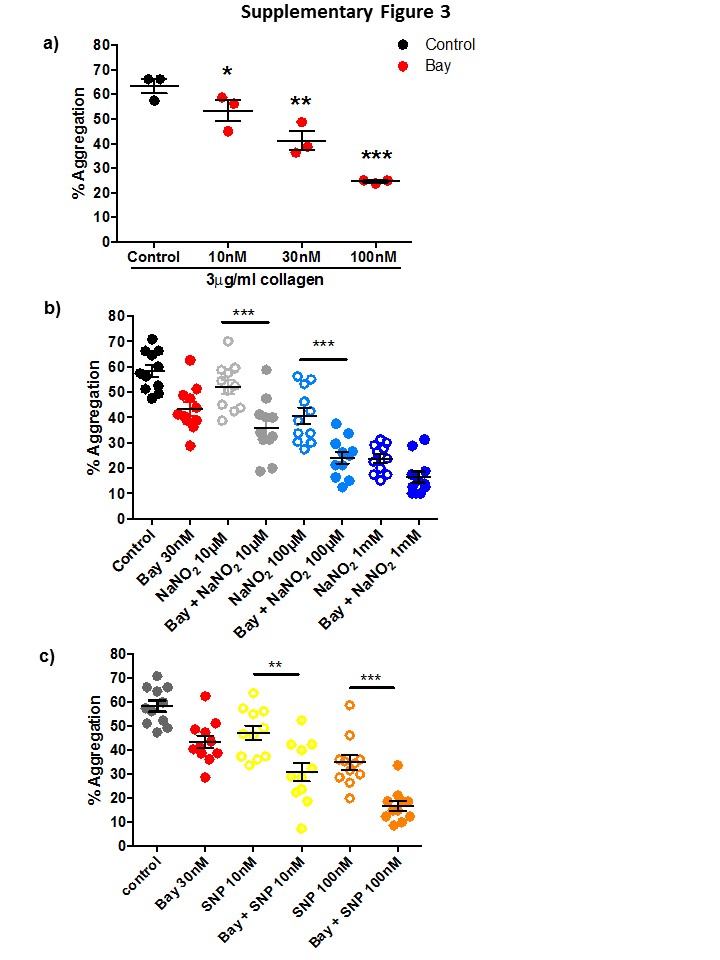

Supplement: Supplementary Data [file cvy087_supp.zip › cvy087-suppl_data/Supplementary Figure_3.jpg]
